# The Interrelationships between Parental Migration, Home Environment, and Early Child Development in Rural China: A Cross-Sectional Study

**DOI:** 10.3390/ijerph17113862

**Published:** 2020-05-29

**Authors:** Jingdong Zhong, Lena Kuhn, Tianyi Wang, Chengfang Liu, Renfu Luo

**Affiliations:** 1 School of Economics, Peking University, Beijing 100871, China; jdzhong@pku.edu.cn; 2 Leibniz Institute of Agricultural Development in Transition Economics, 06120 Halle, Germany; Kuhn@iamo.de; 3 China Center for Agricultural Policy, School of Advanced Agricultural Sciences, Peking University, Beijing 100871, China; tianyi_ccap@pku.edu.cn (T.W.); cfliu.ccap@pku.edu.cn (C.L.)

**Keywords:** parental migration, home environment, early child development

## Abstract

A growing body of literature is providing evidence of a negative association between parental migration and child development. Meanwhile, the chain of relationships between parental migration, home environment, and early child development has not yet been well documented in China. This paper investigates the interrelationships between parental migration, home environment, and early child development in an undeveloped area of western rural China. In total, 444 households were included in the study. Bayley Scales of Infant Development version III (BSID-III), Home Observation Measurement of the Environment (HOME), and a socioeconomic questionnaire, were used to measure children’s development outcomes, home environment, and socioeconomic characteristics in sample households. A mediation effect model was used to estimate the interrelationships between parental migration, home environment, and child development. The results demonstrate that home environment works as a significant mediator, through which parental migration is associated with a 0.07 standard deviation (SD), 0.13 SD, 0.12 SD, and 0.10 SD decline in the child’s cognitive, language, motor, and social-emotional scores, respectively. For future studies, the key findings suggest that interventions aimed at improving the home environments of left-behind children might be necessary in rural China.

## 1. Introduction

Early child development sets the foundations for an individual’s lifelong psychological and physiological well-being. However, as estimated by WHO, 43% of children in low-middle income countries do not reach their full development potential [1]. A recent study has revealed drastic developmental delays in rural China: 49% of the children were delayed in cognition; 52% were delayed in language; 30% were delayed in motor; and 53% were delayed in social-emotion [2], problems that were identified as the root of a potential middle-income trap [3].

A stimulating home environment is considered to contribute to healthy early child development [4,5,6,7,8,9,10]. It comprises the provision of stimulating and learning experiences and a positive emotional climate supporting learning and stimulation processes, as well as safety and cleanliness of physical surroundings [4]. Across different socioeconomic and cultural settings, many studies have shown that an early literacy environment, reading material and toys can positively influence child development [5,6,7]. In particular, children’s cognitive development seems to be strongly related to the quality of their home environment [8]. Furthermore, home environment was found to influence children’s language ability [9] and their physical health outcomes, for instance obesity [10].

While home environment may influence child development via direct pathways, the mediation effects of home environment on the association between household socioeconomic status (SES) and child development has not yet been estimated in China due to a lack of survey data. In fact, SES has a clear influence on the home environment, including the physical surroundings, as well as parenting practices [11,12,13,14]. Parenting practices stimulate caregiver-child interaction activities, such as singing, reading, and playing with the child [11,12,13].

For Chinese rural households, one prominent SES characteristic is the absence of parents as primary caregivers during early childhood due to extended labor migration to urban areas [15]. Although grandmothers act as primary caregivers in most cases, school engagement and other development aspects of these so-called left-behind children suffer [16]. Cognitive development delays among rural Chinese children are strongly linked to the absence of modern parenting among grandmothers [17]. These findings indicate a negative relationship between parental migration and child development. However, to date, a standard mediation analysis on the role of home environment in the relationships between parental migration and early child development in rural China has been lacking.

The aim of this study is to investigate the interrelationships between parental migration, home environment, and early child development. More specifically, the aims of this study consist of (1) investigating the relationship between parental migration and early child development outcomes; (2) determining whether home environment has a significant mediation effect in the link between them; and (3) identifying the mediation effects of various dimensions of home environment on different development outcomes.

To achieve these aims, the study hypotheses are proposed as followed: (1) parental migration is associated with early child development; (2) home environment has mediation effects on relationships between parental migration and development outcomes; and (3) the mediation effects of various dimensions of home environment vary across development outcomes. This study may contribute to the existing knowledge by revealing the mediation effect of home environment between parental migration and early child development in rural China. This would serve as a starting point for designing targeted policies.

## 2. Methods

### 2.1. Participants

The survey was conducted in one prefecture in the province of Guizhou, a relatively poor province in the southwest of China, ranked 29 out of 34 Chinese provinces in terms of per capita GDP in the year 2016.

The sampling protocol was as follow. First, one county was randomly chosen within the sample prefecture. Second, from a list of all townships in the sample county that was obtained from the local regulatory authority, one township was randomly chosen. The randomization was undertaken in both stages of selection by using the random number generator. Finally, in each of the township’s nine villages, a list of registered births was obtained from the local official; according to the sample size calculated for a randomized controlled trial, all households with children aged 6–24 months were enrolled.

The ethical assessment of the study (No. IRB00001052-17056) was approved by the Peking University Institutional Review Board (PU IRB), Beijing, China. The purpose of the study was explained to the parents or the caregivers of all of the children, and then verbal informed consent was obtained from them.

### 2.2. Data Collection

**Figure A1:**
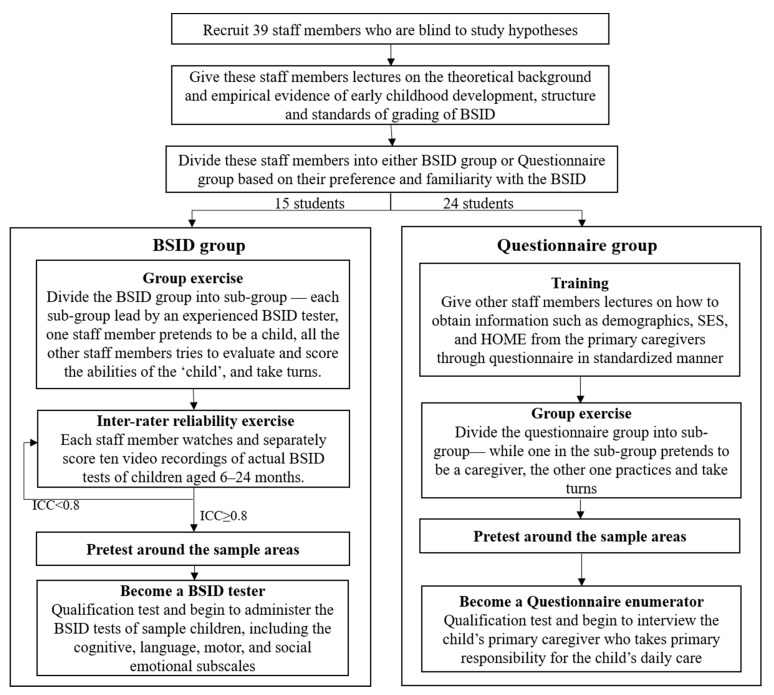
Flowchart of training staff members.

Prior to the 2017 fieldwork, 39 college students were trained as staff members to assess the developments of sample children, and to interview the child’s primary caregiver who takes primary responsibility for the child’s daily care. All staff members were unaware of the study hypotheses to prevent any bias during the interviews. The 39 staff members were divided into two groups. As shown in Figure A1, one group (15 staff members) was trained step by step to build the proficiency in the Bayley Scales of Infant Development (BSID) test, and the other group (24 staff members) was trained in collecting information on home environment, demographic characteristics and socioeconomic status. Both groups were trained intensively for eight hours per day for seven consecutive days to ensure that they consistently understood the survey and were able to administer it in a standardized way.

During and after the fieldwork, quality control approaches such as a triple-check system (self-check, intragroup check, team leader check) and carefully designed logic jumps were implemented. First, at the proposal stage, the research protocols and questionnaires were carefully developed. Second, pretesting was conducted around the sample areas to offer the team an opportunity to identify important questions for the study and to revise the logic jumps in the questionnaire. Additionally, during the process of data collection, self-check and crosschecking was conducted at the staff member level before the staff members left the sample village, and special investigations were also routinely conducted by the team. In the fieldwork, trained staff members made a 90–120 min long home visit when the primary caregiver and the child were both present.

In the survey, the following instruments were used:

(1) Demographic and socioeconomic survey. This questionnaire was administered to the primary caregivers in each sample household to collect data on detailed demographic and socioeconomic characteristics, including the child’s gender, the child’s age in months, whether the child had a low birth weight, whether the child was premature, the primary caregiver’s age, the primary caregiver’s education, and whether the child’s parents are at home or migrant workers. If both of the child’s parents were migrant workers, the household was labelled as a “parental migration” household. This questionnaire had been used previously and validated in the Chinese population [2].
(2) Bayley Scales of Infant Development version III (BSID-III). Designed by Bayley [18], the BSID-III test is a standardized instrument to assess a child ‘s development during age 0–3 [19]. To allow for a standardized development assessment, the test takes into account each child’s age and whether the child was premature. Four scales are included in the test to evaluate the child’s cognitive, language, motor, and social-emotional development. These scales have been formally adapted to the language and the environment context, and have been used in a couple of studies in rural areas of China [2]. In addition, the scales have been checked for reliability in the Chinese population, and the reliability coefficents are larger than 0.8 for all of these scales [20], which indicates that the reliability is adequate [21]. The cognitive, language, and motor scores depend on the number of of tasks that the child could successfully complete [19], while the social-emotional score is based on the caregivers’ responses to a series of questions developed from the Greenspan Social-Emotional Growth Chart [22]. Higher scores correspond to better development. The trained staff members used a standardized set of play materials and a detailed scoring sheet to administer the test. The caregiver was present but not allowed to help the child in completing the tasks.
(3) Home Observation Measurement of the Environment (HOME). Designed by Bradley et al. [23], the HOME inventory is a widely used tool to access the quality of home environment for children. The primary caregiver was asked to complete a questionnaire that was adapted from the HOME in the context of the Chinese language and environment to assess the home environment. No item was removed from the original tool. As shown in Table A1, the questionnaire includes 43 binary-choice items in six subscales: caregiver responsivity, acceptance, organization, learning materials, caregiver involvement, and variety of stimulation. The Cronbach’s alpha reliability coefficients for the scales are between 0.7 and 0.9, indicating that the internal consistency is adequate in the sample [21]. The scores of the relevant items was summed up to calculate the total score of the scales. The higher total score is, the quality of home environment is better.

### 2.3. Statistical Analysis

In order to examine the mediation effect of home environment on the relationship between parental migration and a child’s development outcomes, the following mediation model was used:

(1) $$bayley_{i}=\alpha +\beta_{1}pmigrant_{i}+\beta_{2}home_{i}+\gamma X_{i}+u_{j}+\varepsilon_{i}$$

(2) $$home_{i}=\alpha +\beta_{3}pmigrant_{i}+\gamma X_{i}+u_{j}+\varepsilon_{i}$$

where $bayley_{i}$ is child *i*’s cognitive, language, motor, and social-emotional score in the BSID-III test; $pmigrant_{i}$ is the dummy denoting parental migration; $home_{i}$ is the total score of HOME in household *i*; $X_{i}$ refers to covariates on other socioeconomic characteristics, including the child’s gender, the child’s age, whether the child had a low birth weight, whether the child was premature, the caregiver’s age, and the caregiver’s education; $u_{j}$ refers to the village fixed effects; and $\varepsilon_{i}$ is the random error term. The standard errors were clustered at the village level. $\beta_{1}$ indicates the direct effect of parental migration on the child’s development outcomes, and the product term $\beta_{2}\beta_{3}$ indicates the indirect effect through the home environment. Statistical analyses were performed by using the Stata 15.0 software and the statistical program for mediation effect modeling (medeff package, the version released in 2011).

Following Preacher and Hayes [24], the bootstrap method based on resampling with 1000 replications was used to compute standard errors (S. E.) of the indirect effects. Three types of 95% confidence intervals (CIs), including the percentile CI, the bias-corrected (BC) CI, and the bias-corrected and accelerated (BCa) CI, were calculated to test the statistical significance of the indirect effects [25]. The indirect effect is considered statistically significant if the CI does not contain zero.

## 3. Results

### 3.1. Descriptive Statistics

All 446 households invited to participate agreed to do so. However, two sample households did not finish the interview, culminating in a total of 444 sample households in the analysis.

Table 1 reports the descriptive statistics of the sample. The mean ± SD of children’s cognitive, language, motor, and social-emotional scores in the sample are 94.5 ± 16.2, 89.8 ± 13.7, 95.4 ± 15.8, and 85.4 ± 11.9, respectively. In a healthy children population, the mean scores are expected to be 105 ± 9.6, 109 ± 12.3, 107 ± 14, and 100 ± 15 for the cognitive, language, motor, and social-emotional scales [2], respectively.

In terms of socioeconomic characteristics, about 50% of children were “left behind” as both of their parents migrate out to urban areas. A total of 60% of the children were male. Children were slightly less than 15 months old on average. Seven percent of children had low birth weight. Nine percent of children were born prematurely. The caregivers were around 39 years old, and completed less than five years of schooling on average.

### 3.2. The Mediation Effects of Home Environment

Table 2 reports the ordinary least square (OLS) estimates of the correlations between parental migration, home environment, and child development indicated by Equations (1) and (2). As shown in Columns (1)–(4), the direct effects of parental migration on child’s language development are significantly negative at the 5% significance level with an effect size of 0.20 standard deviation (SD). Meanwhile, the direct effects on child’s cognitive, motor, and social-emotional development are not statistically significant at the 10% level.

Home environment, however, is significantly and positively correlated with the child’s cognitive, language, motor, and social-emotional development. A one SD increase in the HOME score corresponds to a 0.11 SD, 0.19 SD, 0.17 SD, and 0.15 SD increase in the child’s cognitive, language, motor, and social-emotional score, respectively. As shown in Column (5), parental migration is significantly and negatively correlated with home environment at the 1% level. On average, households with parental migration exhibit 0.70 SD lower home environment than those with parents at home as child’s primary caregivers. The results suggest that mediation effects of home environment might exist in the relationship between parental migration and child development.

As shown in Table 3, the point estimates of indirect effects through home environment are significantly smaller than zero. Specifically, the effect sizes of indirect effects on the child’s cognitive, language, motor, and social-emotional development are 0.07 SD, 0.13 SD, 0.12 SD, 0.10 SD, respectively. The indirect effects on the four development outcomes appear to be noticeably different in terms of effect size. However, the 95% CIs of the estimated indirect effects overlap, which indicates that the differences in effect size of indirect effect across development outcomes are not statistically significant. Furthermore, the three 95% CIs in Columns (3)–(5) strongly suggest that the indirect effects through home environment are statistically significant. That is, parental migration could worsen the quality of home environment and, in turn, hinder a child’s early development.

### 3.3. Hetereogeneity

Given the multifactorial measure of home environment, investigation of subscales as strong mediators was then conducted. The HOME six subscale scores were used as mediators, and the estimates of indirect effects through these mediators are reported in Table 4, Table 5, Table 6 and Table 7, respectively.

Table 4 reports estimates of indirect effects of parental migration on cognitive development through the mediators. Learning material (such as stroller, learning facilitators, and toys listed in Table A1) is the only significant mediator, whose three 95% CIs do not contain zero. Through learning material, parental migration is accompanied by a 0.08 SD decline in the child’s cognitive score at the 1% level. By contrast, the mediation effects of other subscales are not statistically significant.

Table 5 reports estimates of indirect effects of parental migration on language development through the mediators. Of the six subscales, three are significant mediators. Organization of stimulating activities (such as taking child on an outing, having a special place for child’s toys and treasures, and keeping child’s play environment safe) is the strongest mediator with an indirect effect of −0.11 SD and significant at the 1% level. The indirect effects mediated by learning material and variety of stimulation are both equal to −0.07 SD and significant at the 5% level.

Table 6 reports estimates of indirect effects of parental migration on motor development through the mediators. The 95% CIs strongly suggest that organization of stimulating activities, learning material, and caregiver responsivity are significant mediators, through which indirect effects are −0.13 SD, −0.10 SD, and −0.02 SD at the 1%, 1%, and 5% level, respectively, while other subscales are not significant mediators.

Table 7 reports estimates of indirect effects of parental migration on social-emotional development through the mediators. Only two subscales are significant mediators: learning material and caregiver involvement, through which parental migration is associated with 0.06 SD and 0.05 SD decline in the child’s social-emotional score, respectively, at the 5% level. The mediation effects of other subscales are not statistically significant.

In short, the mediation effects of home environment in the relationships between parental migration and early child development vary across different subscales. In contrast to other subscales, learning material was the strongest mediator for child’s cognitive and social-emotional development, while organization of stimulating activities was the most important mediator for child’s language and motor development.

## 4. Discussion

This paper explored the interrelationships between parental migration, home environment, and child’s early development outcomes in rural China. Parental migration was negatively and significantly associated with child’s language development, but not directly correlated with child’s cognitive, motor, and social-emotional development. In addition, home environment was the significant channel across parental migration and child’s development outcomes. Furthermore, different dimensions of home environment played different roles in the links between parental migration and development outcomes. Learning material was a strong mediator, which had significant mediation effects on a child’s four outcomes, while organization of stimulating activities worked as a significant mediator only for a child’s language and motor development.

Given that early child development exerts vital impacts not only on one’s lifelong welfare, such as physical health [26,27], earnings [28,29], social mobility [30], and other wellbeing in the adulthood [31,32], but also on the country’s long-term economic growth [33], the main finding of this paper indicates that the large percentage of parental migration could be detrimental to child development in rural China and, thus, might explain the developmental delays of children in these areas, at least partly.

In addition, the findings strongly suggest the mediator role of home environment in the relationship between parental migration and child development. Households without parents present as primary caregivers provide a poorer home environment to children, which is accompanied by worse child development outcomes. This is consistent with a growing body of studies that have summarized rich evidence about the importance of the home environment, i.e., that a high-quality early home environment significantly contributes to the better abilities, personality, and behaviors of children [34,35].

Furthermore, the finding shows the heterogeneity across various dimensions of the home environment. On the one hand, in terms of material investments, rural households invest very few learning materials in child, which in turn leads to negative impacts on children’s development outcomes [36,37]. In contrast to households in which parents are present to act as primary caregivers, households with parental migration invest in less learning materials for children. The decrease in material investments is a strong channel linked to inferior development outcomes including cognition, language, motor, and social-emotion skills. On the other hand, in terms of time investments, stimulating caregiver-child interactions are productive inputs for child outcomes, as documented by previous studies in developed countries [38,39] and in rural areas of China [17,40,41]. Households with parental migration perform worse in the organization of the stimulating activities, which is also negatively correlated with the child’s language and motor development.

In some provinces of China, such as Shaanxi [42], Hebei, and Yunnan Provinces [43], several parenting intervention programs have been initiated to improve early child development of rural children. These programs have seen an increase in children’s development outcomes by 0.23–0.27 SD in treatment households. The findings of this paper indicate that the improvement in home environment could be a key channel between parenting intervention and children’s development outcomes. Furthermore, the findings suggest the necessary implications in terms of targets on home environment for future interventions.

A strength of the study was to recruit individuals from rural households of western China. As shown in Wang et al. [2], in the four major subpopulations of rural China, 53% of the children were male on average; children were around 16 months old on average; and 6% of children were born prematurely. The demographic characteristics of the sample children in this paper are comparable to the populations of other communities of rural China. However, the study also faces some limitations. The data was collected in western rural China; thus the conclusions cannot simply be applied to other developing contexts. Moreover, although OLS estimates are indeed useful to understand the interrelationships of parental migration, home environment, and child development, it is worth noting that the results do not state causal effects. Furthermore, even though having a considerable number of staff members to collect data was also a limitation of the study, they were well trained through a rigorous process.

## 5. Conclusions

In conclusion, this study demonstrated the existence of interrelationships between parental migration, home environment, and development outcomes among children aged 0–3 years old in rural China. It showed that the home environment mediates the link between parental migration and early child development. These findings deserve attention from policy makers, as they imply that targeted interventions to improve the home environment of these left-behind children, especially aimed at increasing learning materials in the households and improving organization of stimulating activities, might be necessary to foster human capital development in rural areas. However, further investigation is necessary to explore the causal links between parental migration, home environment, and early child development.

## Figures and Tables

**Table 1:** Descriptive statistics.

Variable	Definition	Mean ± SD	Min	Max
**Dependent variable**				
*cog*	cognitive score	94.5 ± 16.2	55	140
*lang*	language score	89.8 ± 13.7	50	132
*motor*	motor score	95.4 ± 15.8	46	145
*soemo*	social-emotional score	85.4 ± 11.9	55	130
**Independent variable**				
*pmigrant*	dummy, 1 = parental migration	0.5 ± 0.5	0	1
**Intermediate variable**				
*home*	total score of HOME	25.5 ± 5.4	10	39
**Covariates**				
*male*	dummy, 1 = male	0.6 ± 0.5	0	1
*month*	child’s age in months	14.6 ± 5.5	4	26
*lbw*	dummy, 1 = child has low birth weight	0.07 ± 0.3	0	1
*premature*	dummy, 1 = child is premature	0.09 ± 0.3	0	1
*cage*	age of caregiver	39.0 ± 15.1	15	85
*cedu*	education of caregiver	4.4 ± 4.2	0	16

**Table 2:** Correlations between parental migration, home environment, and child development.

	*Cog*	*Lang*	*Motor*	*Soemo*	*Home*
	(1)	(2)	(3)	(4)	(5)
*Pmigrant*	−0.13 (0.10)	−0.20 ** (0.09)	−0.13 (0.12)	−0.13 (0.12)	−0.70 *** (0.14)
*Home*	0.11 ** (0.05)	0.19 *** (0.04)	0.17 *** (0.04)	0.15 *** (0.02)	
*Male*	0.20 (0.13)	−0.09 (0.12)	0.07 (0.11)	−0.04 (0.12)	0.02 (0.09)
*Month*	−0.03 ** (0.01)	−0.03 * (0.01)	0.05 *** (0.01)	0.01 (0.01)	0.005 (0.006)
*Lbw*	−0.32 * (0.16)	−0.23 (0.15)	−0.26 (0.20)	−0.34 ** (0.13)	−0.33 ** (0.11)
*Premature*	0.06 (0.17)	0.02 (0.19)	0.09 (0.19)	0.20 (0.17)	−0.30 (0.16)
*Cage*	−0.0001 (0.006)	0.004 (0.005)	0.006 (0.004)	0.005 (0.005)	0.001 (0.003)
*Cedu*	0.04 *** (0.01)	0.05 ** (0.02)	0.05 ** (0.02)	0.04 *** (0.01)	0.07 *** (0.02)
Village FE	Yes	Yes	Yes	Yes	Yes
Observations	444	444	444	444	444

Notes: (i) In Columns (1)–(4), the independent variables are parental migration (*pmigrant*) and home environment (*home*), and the dependent variables are child’s cognitive score (*cog*), language score (*lang*), motor score (*motor*), and social-emotional score (*soemo*), respectively. In Column (5), the independent variable is parental migration (*pmigrant*), and the dependent variable is home environment (*home*). Definitions of covariates are in Table 1. (ii) Standardized coefficients (in terms of changes in SD) are reported in the table, and robust standard errors clustered at the village level are presented in parentheses. (iii) *** *p* < 0.01, ** *p* < 0.05, * *p* < 0.1.

**Table 3:** Estimates of the indirect effects of parental migration on child development through home environment.

Indirect Effect	Point Estimate	Bootstrap S. E.	95% CI (Percentile)	95% CI (BC)	95% CI (BCa)
	(1)	(2)	(3)	(4)	(5)
*Pmigrant* on *cog* through *home*	−0.07 **	0.03	[−0.14, −0.01]	[−0.14, −0.02]	[−0.14, −0.02]
*Pmigrant* on *lang* through *home*	−0.13 ***	0.03	[−0.18, −0.06]	[−0.20, −0.07]	[−0.20, −0.07]
*Pmigrant* on *motor* through *home*	−0.12 ***	0.05	[−0.23, −0.04]	[−0.24, −0.05]	[−0.24, −0.05]
*Pmigrant* on *soemo* through *home*	−0.10 ***	0.03	[−0.16, −0.04]	[−0.19, −0.06]	[−0.19, −0.05]

Notes: (i) The independent variable is parental migration (*pmigrant*), and the dependent variables are child’s cognitive score (*cog*), language score (*lang*), motor score (*motor*), and social-emotional score (*soemo*), respectively. The mediator is home environment (*home*). (ii) Point estimates reported in Column (1) are changes in SD. (iii) Bootstrap standard errors reported in Column (2) are based on resampling with 1000 replications. (iv) *** *p* < 0.01, ** *p* < 0.05.

**Table 4:** Estimates of indirect effects of parental migration on cognitive development through different dimensions of home environment.

Indirect Effect	Point Estimate	Bootstrap S. E.	95% CI (Percentile)	95% CI (BC)	95% CI (BCa)
	(1)	(2)	(3)	(4)	(5)
*Pmigrant* on *cog* through *response*	0.004	0.01	[−0.02, 0.02]	[−0.02, 0.03]	[−0.02, 0.03]
*Pmigrant* on *cog* through *accept*	−0.005	0.01	[−0.02, 0.01]	[−0.03, 0.01]	[−0.03, 0.01]
*Pmigrant* on *cog* through *organ*	−0.06	0.05	[−0.16, 0.03]	[−0.16, 0.03]	[−0.16, 0.03]
*Pmigrant* on *cog* through *learnm*	−0.08 ***	0.03	[−0.13, −0.02]	[−0.14, −0.04]	[−0.14, −0.03]
*Pmigrant* on *cog* through *involv*	−0.007	0.02	[−0.05, 0.03]	[−0.05, 0.03]	[−0.05, 0.03]
*Pmigrant* on *cog* through *variety*	−0.05	0.04	[−0.12, 0.02]	[−0.12, 0.02]	[−0.12, 0.02]

Notes: (i) The independent variable is parental migration (*pmigrant*), and the dependent variables is child’s cognitive score (*cog*). The mediators are the HOME six subscales: caregiver responsivity (*response*), acceptance (*accept*), organization (*organ*), learning materials (*learnm*), caregiver involvement (*involv*), and variety of stimulation (*variety*). (ii) Point estimates reported in Column (1) are changes in SD. (iii) Bootstrap standard errors reported in Column (2) are based on resampling with 1000 replications. (iv) *** *p* < 0.01.

**Table 5:** Estimates of indirect effects of parental migration on language development through different dimensions of home environment.

Indirect Effect	Point Estimate	Bootstrap S. E.	95% CI (Percentile)	95% CI (BC)	95% CI (BCa)
	(1)	(2)	(3)	(4)	(5)
*Pmigrant* on *lang* through *response*	−0.02	0.02	[−0.05, 0.002]	[−0.06, 0.001]	[−0.06, 0.001]
*Pmigrant* on *lang* through *accept*	−0.003	0.007	[−0.02, 0.01]	[−0.02, 0.01]	[−0.02, 0.01]
*Pmigrant* on *lang* through *organ*	−0.11 ***	0.04	[−0.19, −0.04]	[−0.18, −0.03]	[−0.18, −0.03]
*Pmigrant* on *lang* through *learnm*	−0.07 **	0.03	[−0.14, −0.03]	[−0.12, −0.02]	[−0.12, −0.02]
*Pmigrant* on *lang* through *involv*	−0.02	0.02	[−0.05, 0.02]	[−0.05, 0.01]	[−0.05, 0.01]
*Pmigrant* on *lang* through *variety*	−0.07 **	0.03	[−0.14, −0.005]	[−0.15, −0.01]	[−0.15, −0.01]

Notes: (i) The dependent variable is child’s language score (*lang*). Others are the same as Table 4. (ii) *** *p* < 0.01, ** *p* < 0.05.

**Table 6:** Estimates of indirect effects of parental migration on motor development through different dimensions of home environment.

Indirect Effect	Point Estimate	Bootstrap S. E.	95% CI (Percentile)	95% CI (BC)	95% CI (BCa)
	(1)	(2)	(3)	(4)	(5)
*Pmigrant* on *motor* through *response*	−0.02 **	0.01	[−0.05, −0.001]	[−0.06, −0.002]	[−0.06, −0.002]
*Pmigrant* on *motor* through *accept*	0.004	0.009	[−0.01, 0.02]	[−0.01, 0.04]	[−0.01, 0.04]
*Pmigrant* on *motor* through *organ*	−0.13 ***	0.05	[−0.21, −0.03]	[−0.22, −0.04]	[−0.22, −0.04]
*Pmigrant* on *motor* through *learnm*	−0.10 ***	0.03	[−0.17, −0.04]	[−0.17, −0.04]	[−0.17, −0.04]
*Pmigrant* on *motor* through *involv*	−0.04	0.03	[−0.09, 0.01]	[−0.10, 0.003]	[−0.14, 0.003]
*Pmigrant* on *motor* through *variety*	−0.05	0.04	[−0.14, 0.02]	[−0.14, 0.01]	[−0.14, 0.01]

Notes: (i) The dependent variable is child’s motor score (*motor*). Others are the same as Table 4. (ii) *** *p* < 0.01, ** *p* < 0.05.

**Table 7:** Estimates of indirect effects of parental migration on social-emotional development through different dimensions of home environment.

Indirect Effect	Point Estimate	Bootstrap S. E.	95% CI (Percentile)	95% CI (BC)	95% CI (BCa)
	(1)	(2)	(3)	(4)	(5)
*Pmigrant* on *soemo* through *response*	−0.01	0.01	[−0.05, 0.004]	[−0.05, 0.004]	[−0.05, 0.004]
*Pmigrant* on *soemo* through *accept*	−0.001	0.006	[−0.01, 0.01]	[−0.02, 0.02]	[−0.02, 0.02]
*Pmigrant* on *soemo* through *organ*	−0.06	0.04	[−0.14, 0.02]	[−0.14, 0.02]	[−0.14, 0.02]
*Pmigrant* on *soemo* through *learnm*	−0.06 **	0.03	[−0.12, −0.004]	[−0.12, −0.01]	[−0.12, −0.004]
*Pmigrant* on *soemo* through *involv*	−0.05 **	0.02	[−0.10, −0.006]	[−0.10, −0.006]	[−0.10, −0.006]
*Pmigrant* on *soemo* through *variety*	−0.04	0.04	[−0.11, 0.04]	[−0.10, 0.04]	[−0.10, 0.04]

Notes: (i) The dependent variable is child’s social-emotional score (*soemo*). Others are the same as Table 4. (ii) ** *p* < 0.05.

## Appendix A

**Table A1:** The Home Observation for Measurement of the Environment inventory (infant version).

Subscale	Cronbach’s Alpha
**Responsivity**	
1. Caregiver spontaneously vocalizes to child at least twice.	0.80
2. Caregiver responds verbally to child’s vocalizations or verbalizations.	0.80
3. Caregiver tells child the name of object or person during visit.	0.81
4. Caregiver’s speech is distinct, clear, and audible.	0.80
5. Caregiver initiates verbal interchanges with visitor.	0.79
6. Caregiver converses freely and easily.	0.81
7. Caregiver permits child to engage in messy play.	0.79
8. Caregiver spontaneously praises child at least twice.	0.80
9. Caregiver’s voice conveys positive feelings toward child.	0.83
10. Caregiver caresses or kisses child at least once.	0.80
11. Caregiver responds positively to praise of child offered by visitor.	0.81
**Acceptance**	
12. Caregiver does not shout at child.	0.79
13. Caregiver does not express overt annoyance with or hostility to child.	0.71
14. Caregiver neither slaps nor spanks child during visit.	0.76
15. No more than one instance of physical punishment during past week.	0.72
16. Caregiver does not scold or criticize child during visit.	0.65
17. Caregiver does not interfere with or restrict child three times during visit.	0.77
18. At least ten books are present and visible.	0.84
**Organization**	
19. Caregiver is one of no more than three regular substitutes used for child.	0.69
20. Child is taken on an outing at least once a week.	0.71
21. Child gets out of house at least four times a week.	0.64
22. Caregiver has an emergency medical and/or accident plan.	0.66
23. Child has a special place for toys and treasures.	0.65
24. Child’s play environment is safe.	0.76
**Learning Materials**	
25. Muscle activity toys or equipment.	0.79
26. Push or pull toy.	0.85
27. Stroller or walker, kiddie car, scooter, or tricycle.	0.82
28. Caregiver provides toys for child to play with during the visit.	0.78
29. Cuddly toy or role-playing toys.	0.80
30. Learning facilitators—mobile, table and chair, high-chair, playpen.	0.82
31. Simple eye–hand coordination toys.	0.91
32. Complex eye–hand coordination toys.	0.82
33. Toys for literature and music.	0.87
**Involvement**	
34. Caregiver keeps child in visual range, looks at often.	0.71
35. Caregiver talks to child while doing household work.	0.69
36. Caregiver consciously encourages developmental advance.	0.64
37. Caregiver invests in maturing toys with value via personal attention.	0.65
38. Caregiver structures child’s play periods.	0.75
39. Caregiver provides toys that challenge child to develop new skills.	0.80
**Variety**	
40. Caregiver reads stories to child at least three times weekly.	0.71
41. Child eats at least one meal with caregiver and/or other children.	0.84
42. Caregiver and child visit or receive from neighbors or friends once a month or so.	0.79
43. Child has three or more books of his/her own.	0.65
**Total**	**0.79**
